# Association of serum methionine metabolites with non-alcoholic fatty liver disease: a cross-sectional study

**DOI:** 10.1186/s12986-022-00647-7

**Published:** 2022-03-18

**Authors:** Yi Tang, Xu Chen, Qian Chen, Jinghe Xiao, Jiaxin Mi, Qiannan Liu, Yiran You, Yuming Chen, Wenhua Ling

**Affiliations:** 1 Department of Nutrition, School of Public Health, Sun Yat-Sen University, 74 Zhongshan Rd. 2, Guangzhou, 510080 Guangdong Province People’s Republic of China; 2 Guangdong Provincial Key Laboratory of Food, Nutrition and Health, Guangzhou, 510080 Guangdong Province People’s Republic of China; 3 Department of Epidemiology, School of Public Health, Sun Yat-Sen University, 74 Zhongshan Rd. 2, Guangzhou, 510080 Guangdong Province People’s Republic of China; 4 Department of Cardiology, Sun Yat-Sen Memorial Hospital, Sun Yat-Sen University, Guangzhou, 510120 Guangdong People’s Republic of China

**Keywords:** Methionine metabolites, Non-alcoholic fatty liver disease, Hepatic steatosis, S-adenosylmethionine, S-adenosylhomocysteine, Homocysteine

## Abstract

**Background and project:**

Non-alcoholic fatty liver disease (NAFLD) is viewed as the hepatic manifestation of metabolic syndrome. Methionine metabolites have been linked to metabolic syndrome and its related diseases. Whether serum methionine metabolites levels are associated with NAFLD remains unclear. The study aimed to assess the association between methionine metabolites and NAFLD.

**Methods:**

This cross-sectional study included a total of 2814 individuals aged 40–75 years old. All participants underwent anthropometric measurements, laboratory tests, dietary assessment and abdominal ultrasonography. Multivariable logistic regression analysis was performed to estimate the association of methionine metabolites with NAFLD.

**Results:**

Overall, 1446 with and 1368 without NAFLD were enrolled in this study. Participants with NAFLD had significantly higher serum S-adenosylmethionine (SAM), S-adenosylhomocysteine (SAH) and homocysteine (Hcy) levels, and a lower S-adenosylmethionine/S-adenosylhomocysteine (SAM/SAH) ratio than those without NAFLD (all *P* < 0.001). After adjusting multiple confounders, odds ratios (95% confidence interval) for quartile 4 versus quartile 1 of SAH, Hcy and SAM/SAH ratio were 1.65 (1.27–2.14), 1.63 (1.26–2.12) and 0.63 (0.49–0.83), respectively (all *P* for trend < 0.01). In addition, serum SAH, Hcy levels and SAM/SAH ratio were significantly correlated with the degree of hepatic steatosis (all *P* for trend < 0.001).

**Conclusion:**

Elevated serum SAH, Hcy levels and lower SAM/SAH ratio may be independently associated with the presence of NAFLD in middle-aged and elder Chinese.

**Supplementary Information:**

The online version contains supplementary material available at 10.1186/s12986-022-00647-7.

## Introduction

Non-alcoholic fatty liver disease (NAFLD) has been emerging as the leading chronic liver disease and a significant global health burden gradually, affecting up to 25% of the world population [1]. In China, the prevalence of NAFLD reached 32.9% in 2018. And the incidence has risen substantially over the past decades, from 4.6% in 2011–2013 to 5.2% in 2014–2016 [2]. NAFLD, ranging from isolated hepatic steatosis to non-alcoholic steatohepatitis (NASH) and cirrhosis [3], is viewed as the hepatic manifestation of metabolic syndrome [4]. The progression of NAFLD is often unpredictable and asymptomatic, which makes it easy to be ignored [5]. Therefore, it is necessary to identify new biomarkers in terms of the prediction of the occurrence and development of NAFLD.

Methionine cycle is a key component of one-carbon metabolism which plays an important role in a broad range of metabolic diseases [6]. At this aspect, intermediate metabolites, including S-adenosylmethionine (SAM), S-adenosylhomocysteine (SAH) and homocysteine (Hcy), have been received great attention (Their relationships were shown in Additional file 1: Figure S1). As the direct metabolite of methionine, SAM is the universal methyl donor for cellular methylation. SAH, as a major byproduct of methylation, is the potent feedback inhibitor of SAM-dependent methyltransferases [7]. Elevated plasma SAH concentrations were associated with an increased risk of cardiovascular events in coronary angiography patients [8]. Moreover, S-adenosylmethionine/S-adenosylhomocysteine (SAM/SAH) ratio is considered as the methylation potential or capacity index. A low ratio was associated with increased risks of metabolic diseases such as chronic kidney disease and cardiovascular disease [9, 10]. Hcy is produced from SAH by reversible reaction of SAH hydrolase (SAHH), and thus is intrinsically related to cellular methylation status as well [11]. Hyperhomocysteinemia may be implicated in the development of many metabolic diseases, such as obesity and type 2 diabetes, and a recent study indicated that high plasm Hcy level could aggravate insulin resistance and vascular endothelial dysfunction in patients with type 2 diabetes [12]. Studies have shown that methionine metabolism is highly active in the hepatocytes and serum methionine levels were correlated with hepatic methionine metabolism activity [13–15]. Thus, serum methionine metabolites may be the indicators of liver methionine metabolism [16].

The hallmark of NAFLD is the abnormal accumulation of lipid in the liver [17]. As the major site of both methionine and lipid metabolism, recently, emerging evidence indicates that several methionine metabolites, including SAM, SAH and Hcy, are critical determinants of hepatic lipid levels [18]. Animal experiments showed that methionine metabolism dysregulation might lead to decreased transport of lipids through hypomethylation of phosphatidylcholine (PC) and increased synthesis of lipids via feedbacking activated sterol regulatory element-binding proteins (SREBPs) [19, 20]. Besides, hepatic global DNA hypomethylation was associated with NAFLD, hepatic inflammation and fibrosis [21], and methyl donor supplementation prevented the progression of NAFLD [18]. Although it has been well documented in animal models, there are limited data regarding the association of serum methionine metabolites with the presence and severity of NAFLD in the population. Humans with SAHH deficiency, a rare genetic disease charactered by sharply raising SAH level, possessed mildly active chronic hepatitis with moderate portal fibrosis [22]. At present, a few epidemiology studies investigated whether methionine metabolites are associated with hepatic metabolic diseases, but the results are inconsistent [23, 24]. Therefore, this study sought to evaluate the association between methionine metabolites and NAFLD through a community-based population of middle-aged and elderly Chinese.

## Materials and methods

### Study design and population

**Fig. 1:**
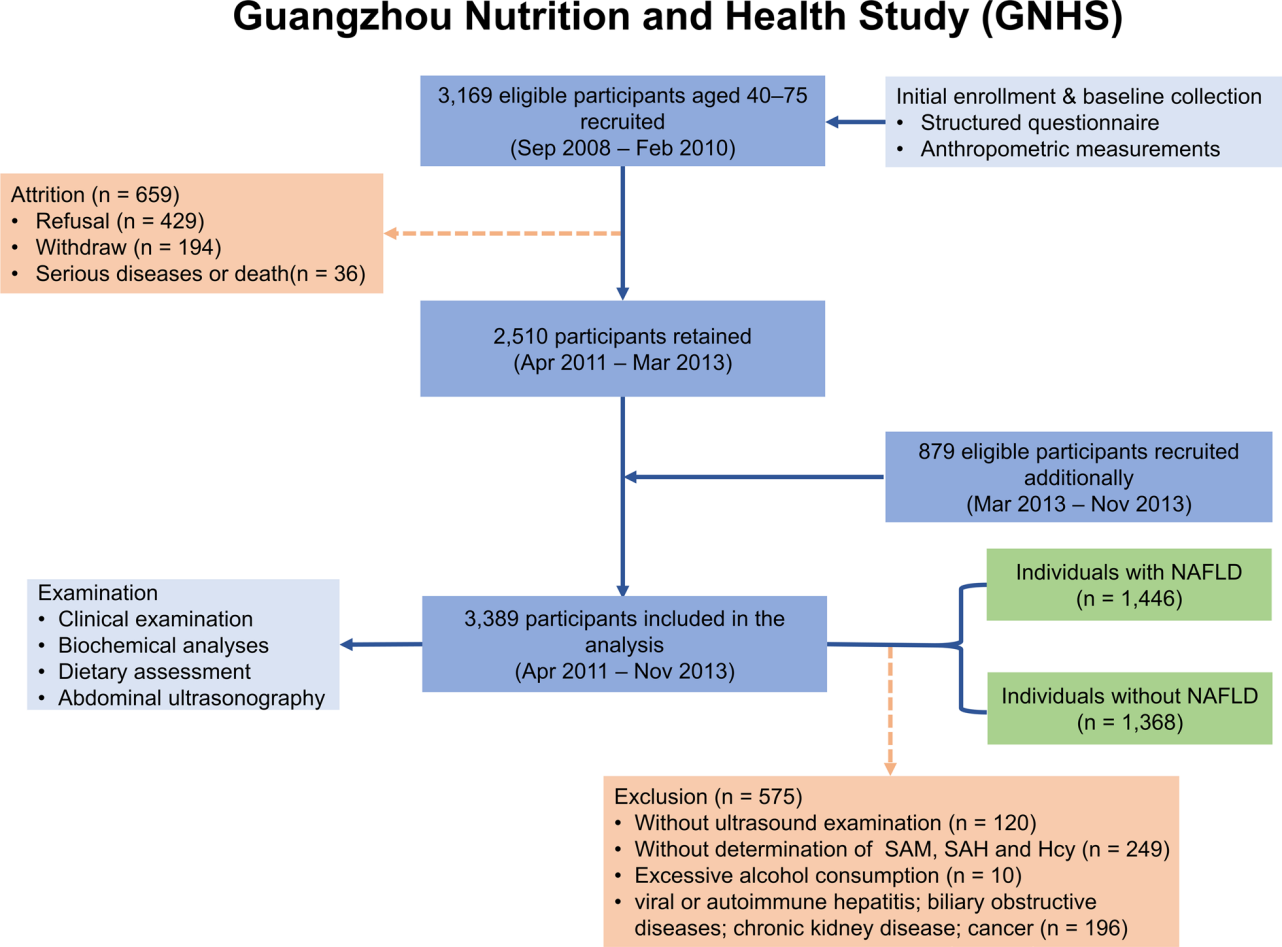
Overview of the study population. *NAFLD* non-alcoholic fatty liver disease, *SAM* S-adenosylmethionine, *SAH* S-adenosylhomocysteine, *Hcy* homocysteine

We conducted a cross-sectional analysis in the Guangzhou Nutrition and Health Study (GNHS), which was performed among the middle-aged and elderly community residents (40–75 years old) in southern China, to evaluate the relationship between the serum methionine metabolites levels and NAFLD. Exclusion criteria included: excess alcohol consumption (≥ 140 g/week in men, ≥ 70 g/week in women); viral or autoimmune hepatitis; drug- or toxin-induced liver diseases; biliary obstructive diseases; genetic liver diseases; chronic kidney disease or renal failure; HIV infection; any type of cancer; current treatment with systemic corticosteroids or anti-inflammatory therapy; or pregnancy [25]. Finally, 2814 participants were included in the analysis (Fig. 1). The study was approved by the Ethics Committee of the School of Public Health at Sun Yat-sen University and all eligible participants signed informed consent.

### Clinical and laboratory measurements

Study participants were invited for a single day visit, in which they underwent comprehensive physical examination, fasting blood test, hepatitis virus test, dietary assessment, abdominal B-scan ultrasonography, a face-to-face interview using a structured questionnaire including demographic characteristics, lifestyle, and habits (e.g., health status, alcohol consumption, smoking, and physical activity) and history of chronic diseases.

Height and weight were obtained with participants wearing light clothes and no shoes. The body mass index (BMI) was calculated as weight in kilograms divided by the square of height in meters (kg/m^2^). The waist-to-hip ratio (WHR) was calculated as waist circumference (cm) divided by hip circumference (cm). The metabolic equivalent (MET) intensity was calculated to estimate daily physical activity using a 24-h physical activity questionnaire. The fat mass of the trunk region was quantified using dual-energy X-ray absorptiometry scans (Discovery W; Hologic Inc., Waltham, MA, USA). Venous blood samples were collected from all the participants in the morning after an overnight fast, and serum was separated and immediately stored within 2 h of collection at − 80 °C until analysis. The samples used for the analysis of biochemical values, including serum fasting glucose, fasting insulin, triglycerides (TG), total cholesterol (TC), high-density lipoprotein cholesterol (HDL-C), low-density lipoprotein cholesterol (LDL-C), aspartate aminotransferase (AST), alanine transaminase (ALT), alkaline phosphatase (ALP), uric acid (UA), and high-sensitivity C-reactive protein (hsCRP). All values were measured by a Hitachi 7600-010 automated analyzer (Hitachi, Tokyo, Japan). Insulin resistance (IR) was evaluated by the homeostasis model assessment (HOMA) calculated as fasting glucose (mM) × fasting insulin (mU/L)/22.5.

Serum methionine metabolites were measured by ultra-high performance liquid chromatography coupled with tandem mass spectrometry (UHPLC-MS/MS, Agilent Technologies, Inc., Santa Clara, CA, USA), as described previously with some differences [26, 27]. In this study, we detected three core serum methionine metabolites in methionine cycle which were related to the methylation, including SAM, SAH and Hcy. Serum samples (50 μL) diluted in ultrapure water (4:1, v/v) were incubated in 37 °C for 15 min after spiking 10 μL DL-dithiothreitol (50 mM) and 10 μL mixture of deuterium-labeled internal standards (^2^H_3_-SAM, 500 nM; ^2^H_4_-SAH, 500 nM; ^2^H_4_-Hcy, 5 μM). Subsequently, 30 μL perchloric acid (1 M) was added to samples for protein precipitation. Then, the samples were centrifuged at 15,000×*g* for 10 min at 4 °C. Finally, the supernatants were filtered by a 0.22 μm membrane. The methionine metabolites were separated through an Acquity BEH C18 column (2.1 × 50 mm; i.d. 1.7 μm) (Waters Corp., Milford, MA, USA), detected by Agilent 1290 Infinity II UHPLC system coupled with Agilent 6410 Triple Quadrupole LC/MS system, and quantified in multiple reaction monitoring mode. The linearity regression coefficients of SAM, SAH and Hcy were more than 0.99, with inter- and intra-assay coefficients of variation less than 10% simultaneously (Additional file 2: Table S1).

### Dietary assessment

Dietary information was collected from a semiquantitative food frequency questionnaire, which requested the participants to state the frequency and amount of food intake during the past 1 years. Intake of methionine, folate and vitamin B_12_ were calculated by multiplying the methionine, folate and vitamin B_12_ composition of each food by the frequency of consumption. The methionine, folate and vitamin B_12_ composition were obtained from the China Food Composition Database.

### Ultrasonography examination

The abdominal ultrasonic examination was performed by the same group of trained and experienced ultrasonographists who were blind to the clinical and laboratory data, using a Doppler sonography (Sonoscape SSI-5500, Shenzhen, China) equipped with a 3.5-MHz probe. Hepatic steatosis was diagnosed by abdominal characteristic echo patterns according to standardized criteria issued by the Chinese Liver Disease Association. NAFLD was defined as diffuse fatty liver following exclusion of alcohol consumption, viral, or autoimmune liver diseases. Moreover, according to the echogenicity, the severity of hepatic steatosis was graded as follows: absent, normal echo pattern; mild, slight and diffuse increase in fine echoes in liver parenchyma with normal visualization of intrahepatic vessel borders and diaphragm; moderate, middling and diffuse enhancement in fine echoes with marginally impaired visualization of intrahepatic vessels and diaphragm; severe, marked elevation in fine echoes with poor or non-visualization of the intrahepatic vessel borders, diaphragm, and posterior right lobe of the liver [28].

There was a good consistency for ultrasound examination against abdominal computed tomography (Spearman r = 0.905, κ = 0.691, and total agreement = 85%, *P* < 0.001) and a good reliability among operators (Spearman r = 0.911, κ = 0.875, and total agreement = 93%, *P* < 0.001).

### Statistical analysis

We computed descriptive statistics of baseline characteristics and serum methionine metabolites concentrations, presented as mean ± standard deviation (SD), median [25th, 75th percentiles], or frequencies with percentages, as appropriate. We evaluated differences in study variables between NAFLD and non-NAFLD participants by the unpaired Student’s t test or Mann–Whitney U test for numerical variables and the Chi-Square test or Fisher’s exact test for categorical data. The correlations between serum methionine metabolites levels and relevant metabolic factors were examined by Spearman correlation analysis and partial correlation analysis adjusting for age and sex. Multivariable logistic regression was performed to analyze the association between methionine metabolites and NAFLD. All participants were classified into quartiles by SAM, SAH, Hcy levels and SAM/SAH ratio. Both univariable and multivariable *P* for trend between quartiles of methionine metabolites and prevalence of NAFLD were calculated by logistic regression analysis. Covariates in the multivariable model, which were chosen according to their clinical importance as well as statistical significance, included age, gender, BMI, WHR, trunk fat ratio, physical activity, current smoking, current drinking, history of hypertension, diabetes, dyslipidemia and heart disease, HOMA-IR, TC, TG, HDL, LDL, AST/ALT ratio, UA, ALP and hsCRP. In order to test whether the results were consistent among different subgroups, we stratified study participants by gender (female vs. male), age (< 65 vs. ≥ 65, years), BMI (no overweight vs. overweight), WHR (no central obesity vs. central obesity), and TG (< 1.7 vs. ≥ 1.7 mM) level from a health perspective and performed logistics regression analysis after adjusting for the same confounding factors. According to the recommended BMI (≥ 24 kg/m^2^) and WHR (≥ 0.9 for female and ≥ 1.0 for male) cut-offs of “Chinese adult overweight and obesity prevention and control guidelines” for determining overweight, central obesity. Moreover, we performed univariate and multivariate comparisons of methionine metabolites levels across hepatic steatosis groups by Kruskal–Wallis one-way analysis of variance (ANOVA) for k samples and analysis of covariate (ANCOVA), respectively. Data manipulation and statistical analyses were performed using SPSS version 25 software (IBM Inc., Chicago, IL). *P* < 0.05 (two-tailed test) was considered statistically significant.

## Results

### Characteristics of the study population

There were eligible 1446 (51.39%) NAFLD and 1368 (48.61%) non-NAFLD participants in the present study. The mean age of the study population was 60.84 ± 5.76 years, and 868 (30.85%) participants were male. The baseline characteristics of participants with and without NAFLD are shown (Table 1). NAFLD participants were much more likely to have higher BMI, WHR, HOMA-IR, TG, higher prevalence rates of hypertension and diabetes and dyslipidemia, and less likely to have higher physical activities, HDL-C and AST/ALT ratio than those without NAFLD. However, there were no differences in LDL-C and TC. Participants with NAFLD had higher concentrations of serum SAM (91.06 vs 85.67 nM), SAH (17.74 vs 14.50 nM) and Hcy (13.61 vs 12.51 μM), and lower SAM/SAH ratio (5.31 vs 6.09) than those without NAFLD (all *P* < 0.001).

**Table 1:** Baseline characteristics of populations with and without NAFLD

Characteristics	Overall	NAFLD (n = 1446)	Non-NAFLD (n = 1368)	*P* value
*Demographics*				
Age (years)	60.84 ± 5.76	61.97 ± 5.45	60.71 ± 6.06	0.221
Sex (male)	868 (30.85)	468 (32.37)	400 (29.24)	0.073
*Anthropometrics*				
BMI (kg/m^2^)	23.61 ± 3.16	25.01 ± 3.00	22.13 ± 2.60	< 0.001
Waist (cm)	84.91 ± 8.80	88.34 ± 8.15	81.29 ± 7.98	< 0.001
WHR	0.92 ± 0.07	0.94 ± 0.06	0.90 ± 0.07	< 0.001
Trunk fat percentage (%)	33.62 ± 6.74	35.65 ± 5.85	31.49 ± 6.95	< 0.001
SBP (mmHg)	125.21 ± 17.93	128.37 ± 17.51	121.88 ± 17.76	< 0.001
DBP (mmHg)	75.57 ± 10.37	77.56 ± 10.16	73.48 ± 10.18	< 0.001
*Blood biochemical indices*				
Fasting glucose (mM)	4.80 [4.39, 5.31]	4.85 [4.43, 5.41]	4.72 [4.33, 5.21]	< 0.001
Fasting insulin (μU/mL)	7.77 [5.39, 11.21]	9.97 [7.00, 13.86]	6.08 [4.42, 8.27]	< 0.001
HOMA-IR	1.67 [1.11, 2.54]	2.18 [1.49, 3.18]	1.30 [0.90, 1.84]	< 0.001
AST/ALT	1.20 [0.96, 1.45]	1.09 [0.87, 1.33]	1.30 [1.08, 1.56]	< 0.001
TC (mM)	5.59 ± 1.05	5.56 ± 1.03	5.63 ± 1.07	0.059
TG (mM)	1.26 [0.90, 1.78]	1.44 [1.04, 2.06]	1.08 [0.81, 1.50]	< 0.001
HDL-C (mM)	1.43 ± 0.41	1.32 ± 0.36	1.55 ± 0.43	< 0.001
LDL-C (mM)	3.60 ± 0.91	3.62 ± 0.90	3.57 ± 0.91	0.140
UA (μM)	350.50 ± 84.32	364.68 ± 83.31	335.49 ± 82.80	< 0.001
ALP (U/L)	70.07 [58.87, 83.07]	71.25 [59.47, 84.08]	68.85 [58.23, 81.76]	< 0.001
hsCRP (mg/L)	0.96 [0.57, 1.94]	1.19 [0.67, 2.44]	0.77 [0.48, 1.38]	< 0.001
*Serum methionine metabolites*				
SAM (nM)	88.07 [75.04, 110.04]	91.06 [76.90, 111.19]	85.67 [73.62, 108.74]	< 0.001
SAH (nM)	15.70 [11.46, 24.55]	17.74 [12.09, 26.24]	14.50 [10.84, 22.64]	< 0.001
Hcy (μM)	13.06 [11.33, 16.39]	13.61 [11.66, 17.43]	12.51 [10.97, 15.35]	< 0.001
SAM/SAH	5.68 [4.18, 7.20]	5.31 [3.99, 7.01]	6.09 [4.47, 7.42]	< 0.001
*Lifestyle*				
Physical activities (MET/day)	34.05 ± 5.65	33.69 ± 5.55	34.43 ± 5.73	< 0.001
Current smoking	313 (11.12)	164 (11.34)	149 (10.89)	0.704
Current drinking	204 (7.25)	109 (7.54)	95 (6.94)	0.540
Methionine intake (mg/day)	1131.37 ± 283.31	1137.89 ± 290.34	1124.85 ± 276.24	0.223
Folate intake (ng/day)	210.56 ± 83.95	212.12 ± 92.80	208.91 ± 73.44	0.308
Vitamin B_12_ intake (ng/day)	1.43 ± 1.01	1.45 ± 1.15	1.41 ± 0.85	0.257
*History of disease*				
Hypertension	825 (29.35)	518 (35.85)	307 (22.47)	< 0.001
Diabetes	213 (7.58)	127 (8.79)	86 (6.30)	0.019
Dyslipidemia	1099 (39.05)	647 (44.74)	452 (33.04)	< 0.001
Heart diseases	559 (19.91)	302 (20.91)	257 (18.84)	0.330

Values are shown as mean ± standard deviation, median [25th, 75th percentiles] or frequencies (%)

Statistical analysis was performed using *t* test, Mann–Whitney U test, chi-square test

*NAFLD* non-alcoholic fatty liver disease, *BMI* body mass index, *WHR* waist-to-hip ratio, *SBP* systolic blood pressure, *DBP* diastolic blood pressure, *HOMA-IR* homoeostasis model assessment of insulin resistance, *AST* aspartate aminotransferase, *ALT* alanine aminotransferase, *TC* total cholesterol, *TG* triglycerides, *HDL-C* high-density lipoprotein cholesterol, *LDL-C* low-density lipoprotein cholesterol, *UA* uric acid, *ALP* alkaline phosphatase, *hsCRP* high-sensitivity C-reactive protein, *SAM* S-adenosylmethionine, *SAH* S-adenosylhomocysteine, *Hcy* homocysteine, *MET* metabolic equivalent of task

The correlations between methionine metabolites and metabolic related factors were shown in Table 2. Serum SAM was statistically and positively associated with BMI, WHR, HOMA-IR, TG, UA and hsCRP, and statistically and negatively associated with AST/ALT ratio, HDL-C and LDL-C before and after adjustment for age and sex (all *P* < 0.05). Spearman correlation analysis and partial correlation analysis showed that both serum SAH and Hcy were statistically and positively linked to BMI, WHR, HOMA-IR, TG and UA (all *P* < 0.05). Meanwhile, Serum SAH statistically and inversely correlated with AST/ALT ratio and HDL-C, and serum Hcy statistically and negatively related with HDL-C and TC (all *P* < 0.05). On the contrary, the statistically negative correlations between SAM/SAH ratio and BMI, WHR, TG, LDL-C and UA, and the statistically positive correlation between SAM/SAH ratio and HDL were observed (all *P* < 0.05).

**Table 2:** Unadjusted and adjusted Spearman correlation coefficients of methionine metabolites and metabolic relevant factors

Variables	Unadjusted				Adjusted			
	SAM	SAH	Hcy	SAM/SAH	SAM	SAH	Hcy	SAM/SAH
BMI	0.129***	0.172***	0.170***	− 0.101***	0.131***	0.145***	0.117***	− 0.077***
WHR	0.179***	0.152***	0.099***	− 0.055**	0.148***	0.116***	0.045*	− 0.043*
HOMA-IR	0.126***	0.076***	0.098***	0.017	0.085***	0.056***	0.043**	0.010
AST/ALT	− 0.126***	− 0.087***	− 0.037	0.020	− 0.083***	− 0.063**	− 0.028	0.017
TC (mM)	− 0.025	− 0.014	− 0.058**	− 0.004	− 0.015	0.003	− 0.062**	− 0.028
TG (mM)	0.095***	0.155***	0.087***	− 0.108***	0.048*	0.104***	0.049*	− 0.084***
HDL-C (mM)	− 0.131***	− 0.163***	− 0.128***	0.092***	− 0.099***	− 0.144***	− 0.110***	0.093***
LDL-C (mM)	− 0.053**	− 0.002	− 0.023	− 0.038*	− 0.045*	0.010	− 0.023	− 0.046*
UA (μM)	0.188***	0.161***	0.091***	− 0.075***	0.159***	0.112***	0.046*	− 0.049*
hsCRP (mg/L)	0.120***	0.130***	0.091***	− 0.073***	0.066**	0.010	0.022	0.034
SAM (nM)	–	0.527***	0.193***	− 0.002	–	0.476***	0.172***	0.063**
SAH (nM)	0.527***	–	0.416***	− 0.815***	0.476***	–	0.358***	− 0.768***
Hcy (μM)	0.193***	0.416***	–	− 0.341***	0.172***	0.358***	–	− 0.261***
SAM/SAH	− 0.002	− 0.815***	− 0.341***	–	0.063**	− 0.768***	− 0.261***	–

The adjusted associations between serum methionine metabolites levels and several relevant factors were estimated by partial correlation adjusting for age and sex

*P < 0.05; **P < 0.01; ***P < 0.001

### Association between methionine metabolites and NAFLD

Table 3 demonstrated that the proportion of NAFLD tended to increase along with the increased quartiles of SAM, SAH and Hcy levels and the decreased SAM/SAH ratio (all *P* for trend < 0.001). After adjusting potential confounders, the odds of NAFLD increased with increasing SAH level in all models (all *P* for trend < 0.001). The adjusted odds ratios (ORs) (95% confidence interval [CI]) for quartile 4 *vs* quartile 1 of model 1, model 2 and model 3 were 2.46 (1.97–3.08), 1.74 (1.35–2.24) and 1.65 (1.27–2.14), respectively. In addition, serum Hcy level was also positively associated with NAFLD in all models (all *P* for trend < 0.01). The adjusted ORs (95% CI) for quartile 4 *vs* quartile 1 of model 1, model 2 and model 3 were 2.41 (1.93–3.01), 1.73 (1.34–2.23) and 1.63 (1.26–2.12), respectively. Meanwhile, the inverse association of SAM/SAH ratio with NAFLD was observed in all models (all *P* for trend < 0.001). The adjusted ORs (95% CI) in quartile 4 were 0.57 (0.46–0.71) in model 1, 0.62 (0.48–0.80) in model 2, and 0.63 (0.49–0.83) in model 3. However, there was no statistically significant association between SAM level and the odds of NAFLD.

**Table 3:** Adjusted ORs (95% CI) for NAFLD prevalence by quartiles of serum methionine metabolites

	Quartiles of serum methionine metabolites				*P* for trend
	Quartile 1	Quartile 2	Quartile 3	Quartile 4	
SAM (nM)	< 75.05	75.05–88.06	88.07–110.03	> 110.04	
NAFLD (%)	44.52	50.64	56.45	53.91	< 0.001
Model 1	1.00	1.34 (1.08, 1.66)	1.66 (1.33, 2.06)	1.43 (1.15, 1.79)	< 0.001
Model 2	1.00	1.07 (0.83, 1.38)	1.32 (1.03, 1.70)	0.99 (0.77, 1.28)	0.636
Model 3	1.00	1.01 (0.78, 1.31)	1.24 (0.96, 1.61)	0.97 (0.74, 1.26)	0.795
SAH (nM)	< 11.46	11.46–15.69	15.70–24.52	> 24.53	
NAFLD (%)	40.48	49.21	51.28	64.58	< 0.001
Model 1	1.00	1.38 (1.11, 1.71)	1.50 (1.21, 1.87)	2.46 (1.97, 3.08)	< 0.001
Model 2	1.00	1.16 (0.92, 1.51)	1.27 (0.99, 1.63)	1.74 (1.35, 2.24)	< 0.001
Model 3	1.00	1.16 (0.90, 1.50)	1.27 (0.98, 1.65)	1.65 (1.27, 2.14)	< 0.001
Hcy (μM)	< 11.33	11.33–13.05	13.06–16.37	> 16.38	
NAFLD (%)	40.83	49.79	51.07	63.87	< 0.001
Model 1	1.00	1.42 (1.15, 1.76)	1.42 (1.14, 1.76)	2.41 (1.93, 3.01)	< 0.001
Model 2	1.00	1.19 (0.93, 1.53)	1.07 (0.84, 1.38)	1.73 (1.34, 2.23)	< 0.001
Model 3	1.00	1.23 (0.96, 1.60)	1.09 (0.84, 1.41)	1.63 (1.26, 2.12)	0.001
SAM/SAH	< 4.16	4.16–5.74	5.75–7.21	> 7.22	
NAFLD (%)	60.26	53.79	46.75	44.57	< 0.001
Model 1	1.00	0.77 (0.62, 0.96)	0.60 (0.41, 0.75)	0.57 (0.46, 0.71)	< 0.001
Model 2	1.00	0.81 (0.63, 1.04)	0.64 (0.50, 0.82)	0.62 (0.48, 0.80)	< 0.001
Model 3	1.00	0.80 (0.62, 1.03)	0.64 (0.49, 0.83)	0.63 (0.49, 0.83)	< 0.001

Model 1: adjusted for age, sex

Model 2: adjusted as for model 1 plus BMI, WHR, trunk fat ratio, physical activity (MET/day), current smoking, current drinking, history of hypertension, diabetes, dyslipidemia and heart disease;

Model 3: adjusted as for model 2 plus HOMA-IR, TC, TG, HDL, LDL, AST/ALT ratio, UA, ALP and hsCRP

### Subgroup analysis

**Fig. 2:**
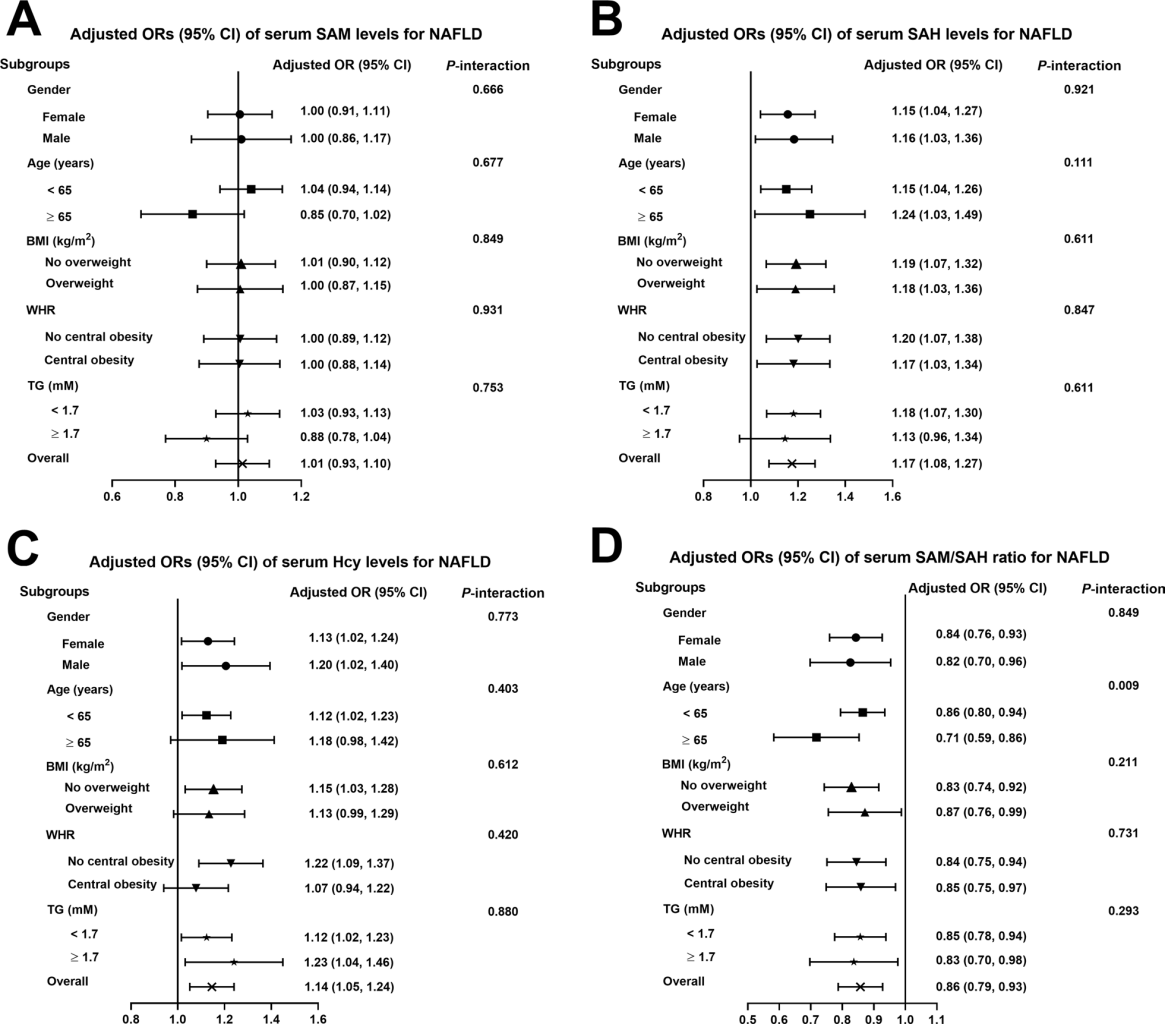
Subgroup analysis for by gender, age, BMI, HOMA-IR and TG using multivariable logistic regression. The data are shown as the ORs (95% CI) in each quartile of serum **A** Hcy, **B** SAH, **C** SAM, **D** SAM/SAH levels for NAFLD

Additionally, in subgroup analysis, the positive associations between SAH and the presence of NAFLD were consistent in all subgroups analyzed except for TG stratification. The positive associations between Hcy and NAFLD were detected in younger participants and those without overweight and central obesity (all *P* < 0.05). There were no significant interactions between quartiles of SAH and Hcy and stratified factors in all stratifications, including gender, age, BMI, WHR and TG (all *P* for interaction > 0.05). Besides, subgroup analysis also showed an inconsistently inverse association between SAM/SAH ratio and NAFLD, with significant interactions by age categories (*P* for interaction < 0.01). However, there were no associations between SAM and NAFLD in all subgroups analyzed (Fig. 2).

### Correlation of methionine metabolites and degree of hepatic steatosis

**Fig. 3:**
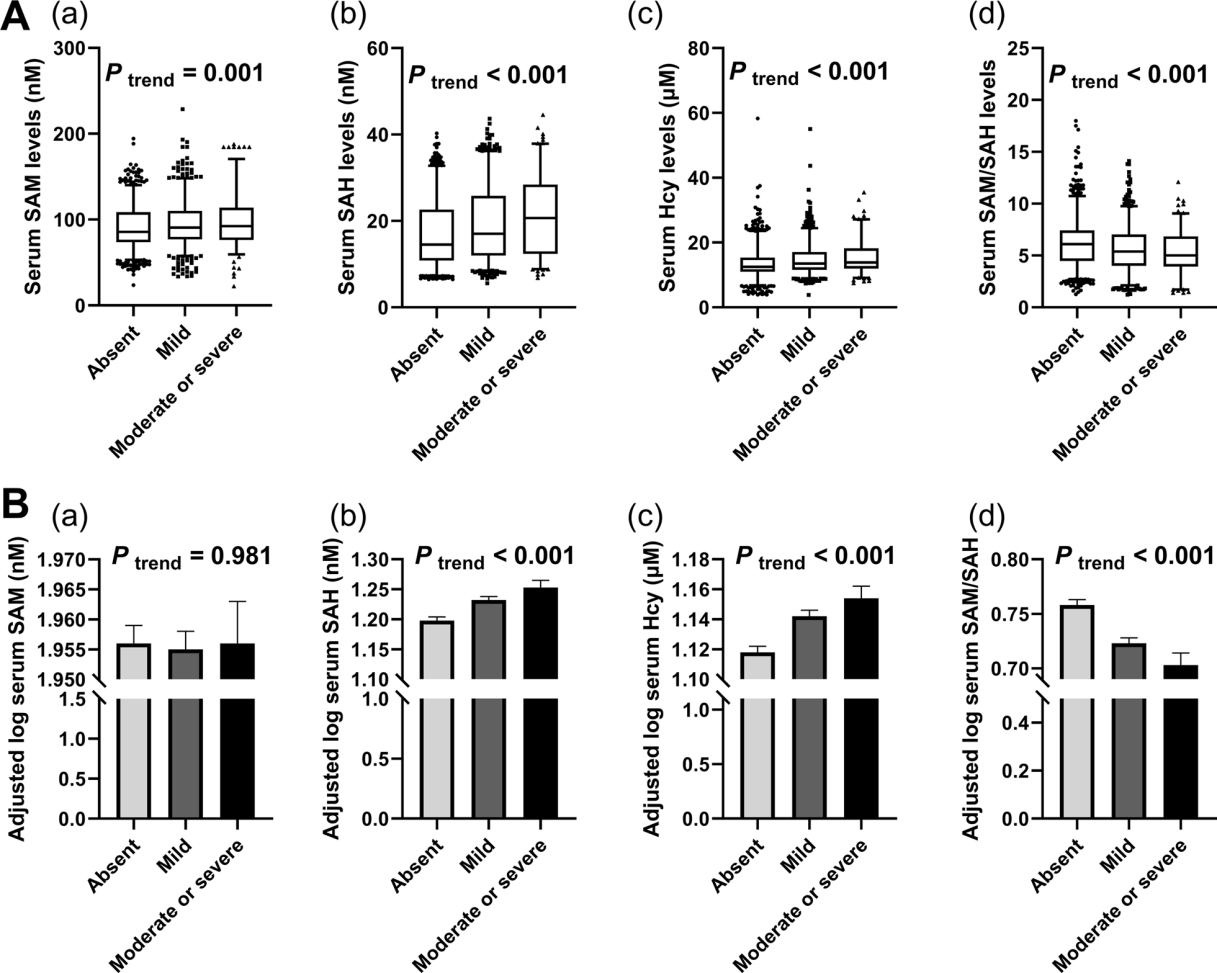
Correlation of methionine metabolites and degree of hepatic steatosis. **A** Univariate and **B** multivariate. **A** Univariate *P* values are calculated by were analyzed by Kruskal–Wallis one-way ANOVA for k samples. **B** serum (a) SAM, (b) SAH, (c) Hcy, (d) SAH/SAM ratio (log-transformed) were adjusted for age, gender, BMI, WHR, trunk fat ratio, physical activity, current smoking, current drinking, history of hypertension, diabetes, dyslipidemia and heart disease, HOMA-IR, AST/ALT ratio, TC, TG, HDL, LDL, UA, ALP and hsCRP. Multivariate *P* values are analyzed by ANCOVA

Next, we analyzed whether concentrations of serum methionine metabolites linked with the severity of hepatic steatosis which was defined semi-quantitatively and rated as absent, mild, moderate or severe based on ultrasonographic features. As shown in Fig. 3A, the concentrations of SAM, SAH and Hcy significantly increased with the degree of hepatic steatosis (all *P* for trend < 0.01). Conversely, SAM/SAH ratio significantly decreased as the degree of hepatic steatosis increased in all participants (*P* for trend < 0.001). Also, in the light of the results of ANCOVA (Fig. 3B), comparable associations continued to be observed in serum SAH, Hcy levels and SAM/SAH ratio (log-transformed) after adjusting for relevant confounders, including demographic characteristics, physical fitness indexes, history of diseases, lifestyle and biochemical indices (all *P* for trend < 0.001). However, serum SAM level (log-transformed) no longer increased with the degree of hepatic steatosis.

## Discussion

In this observational study, we found that subjects with NAFLD had higher serum SAH and Hcy levels and lower SAM/SAH ratio than those without NAFLD in middle-aged and elderly Chinese. The results of the current study demonstrated that SAH, Hcy levels and SAM/SAH ratio may be closely associated with NAFLD, which was consistently observed in both univariable and multivariable adjusted analyses. Furthermore, with the degree of hepatic steatosis increasing, serum SAH and Hcy concentrations tended to increase and SAM/SAH ratio tended to decrease.

Methionine metabolism is involved in a variety of physiological processes, such as the homeostasis of methionine, methylation, biosynthesis of thymidine and purine, and redox defense [29]. Due to the essential role of methionine metabolites, their disorder can result in a series of diseases, including metabolic syndrome [30]. In a cross-sectional study of 1108 Swedish individuals, an independent association between Hcy and serum insulin was closely related to metabolic syndrome [31]. NAFLD is the manifestation of metabolic syndrome in the liver. In the NAFLD population, insulin’s ability to inhibit glucose and LDL production is impaired [32]. Some studies have suggested that subjects with metabolic syndrome frequently exhibited elevated ALT and lipid droplets levels and a higher prevalence of NAFLD [33–35]. In our study, participants with NAFLD had higher metabolic relevant risk factors, higher SAM, SAH, Hcy levels and lower SAM/SAH ratio than those without NAFLD. Parallelly, SAM, SAH and Hcy was positively correlated with metabolic relevant risk factors, and SAM/ SAH was inversely related with metabolic relevant risk factors. These findings hinted that methionine metabolites were associated with metabolic disorders, which may be closely linked to the development of NAFLD.

In the current circumstances, a few studies have explored the association between methionine metabolites and NAFLD, and the conclusions were still controversial. For instance, Dai et al*.* reported that serum Hcy level were positively associated with NAFLD, particularly in female, obese or non-smoking adults [23]. But some studies held contrary opinions that Hcy was independently inversely associated with NAFLD and NASH [24, 36, 37]. The possible reasons for these inconsistencies may be as follows. First of all, the sample size of the population was insufficient, resulting in potential biases. Secondly, these studies varied in race and disease stage. Last but not least, in these studies, methionine metabolites were often measured by high-performance liquid chromatography with fluorescence detection which was not enough sensitive. Thus, the above limitations might negatively impact the extrapolation of those conclusions. Herein, our study enrolled 2814 middle-aged and elderly community individuals in southern China and investigated a series of methionine metabolites which was detected by UHPLC/MS–MS with higher sensitivity and specificity. And the findings suggested that methionine metabolites may be predictors and risk factors of the presence of NAFLD.

The mechanisms by which elevated serum methionine metabolites such as SAH and Hcy were positively associated with the presence of NAFLD could be as follows. It has been reported that elevated methionine metabolites could disrupt the methylation of a variety of substances, including PC and SREBPs. Hepatic PC is produced via SAM-dependent methylation of phosphatidylethanolamine. PC plays an essential role in maintaining hepatic lipid homeostasis by regulating lipid transport. Decreased PC level weakens the assembly and secretion of lipoproteins especially low-density lipoprotein which impairs lipid excretion and further accelerates intracellular lipid droplet accumulation in the liver [20]. An epidemiological study also showed that participants with hepatic steatosis had 25% less PC in the liver than the normal population [38]. On the other hand, SREBP-1, a transcription factor regulating lipogenesis genes in mammals, can be activated by low PC level in a feedback mechanism. Activated SREBP-1 subsequently upregulates genes involved in lipid biosynthesis, boosting lipid droplet formation and leading to lipid accumulation in hepatocytes as well [19]. Therefore, not only impaired lipid excretion but also increased lipogenesis can promote the occurrence of NAFLD. In addition, higher SAH or Hcy could promote the progression of inflammation in the liver. Arumugam et al. [39] reported that elevated SAH increased the release of pro-inflammatory cytokines from adipocytes. Cristiane et al. [40] showed that elevated Hcy induced oxidative stress and recruited inflammatory cells in the liver of rats.

Notably, there was no association of serum SAM level with NAFLD and no linear trend between SAM and the severity of hepatic steatosis on multivariable analyses. As a dynamic reaction loop, once methionine metabolism is impaired, the reaction process will be blocked to varying degrees and substances in all links will increase. Indeed, in our study, we observed that the serum levels of SAM, SAH and Hcy in the NAFLD population were significantly higher than those without NAFLD. Higher SAM can provide more methyl donors indicating the greater methylation capacity, which is considered to be beneficial to health [41]. However, this beneficial effect may be overshadowed by harmful effects of increased SAH and Hcy. In the study, SAM and SAH levels were higher by 5% and 18% in participants with NAFLD compared with that of those without NAFLD, respectively. The decreased SAM/SAH ratio suggested that the increase of SAM was less than that of SAH in the NAFLD population. Additionally, Lind et al. [42] reported that in the population with metabolic syndrome factors, SAH or Hcy, not SAM, were related to some metabolic characteristics, such as BMI, body fat percentage and AST. Zawada et al. [43] also demonstrated that SAH was more tightly associated with traditional cardiovascular risk factors than SAM in a cardiovascular low-risk population. Given SAH is the strong inhibitor of methylation, we speculate that SAH is a more sensitive indicator to the methylation status compared to SAM, and the decrease of SAM/SAH ratio (methylation capacity index) is mainly due to the increase of SAH level. This conjecture needs further confirmation.

In subgroup analyses, we observed different degree of effects of SAM/SAH ratio on NAFLD participants with different age subgroup (< 65 vs. ≥ 65, *P* for interaction < 0.01). The interaction between SAM/SAH ratio and age may affect NAFLD prevalence. Changes of the DNA methylation level occur with aging, and SAM/SAH ratio as the indicator of DNA methylation capacity might change accordingly, contributing to the development of disorders, such as NAFLD [44]. The associations of serum Hcy level with NAFLD were not consistently the same while positive but not significant associations of serum Hcy level with NAFLD were observed in older (age ≥ 65 years), overweight (BMI ≥ 24 kg/m^2^) and central obesity (WHR ≥ 0.9 for female and ≥ 1.0 for male) subgroups. However, there was no statistically significance of interaction term between serum Hcy and these strata factors. We speculated that older, overweight or central obesity participants tended to change the diet structure and received related treatment, which may be beneficial to their NAFLD.

This study has several limitations. Firstly, the cross-sectional design cannot clarify the causal inference. Whether methionine metabolites are bystanders, causal factors or consequences of NAFLD cannot be answered from the results of this cross-sectional study, and prospective studies are warranted. Secondly, since all study participants had been voluntarily recruited, there is potential for selection bias. Thirdly, NAFLD was diagnosed non-invasively as appropriate for epidemiological studies generally. Nevertheless, compared to liver biopsy, ultrasonography cannot provide histological information to further explore the association between methionine metabolites and NASH. Lastly, all study participants were exclusively Chinese, which restricts the generalizability to other ethnic populations.

## Conclusions

In conclusion, in this cross-sectional study of middle-aged and elderly Chinese, serum SAH and Hcy levels may be positively associated with the risk of NAFLD prevalence, and SAM/SAH ratio may be inversely related to NAFLD.

## Supplementary Information

**Additional file 1**. Figure S1. Methionine metabolism. SAM, S-adenosylmethionine; SAH, S-adenosylhomocysteine; Hcy, homocysteine; ATP, adenosine triphosphate; MAT, methionine adenosyltransferase; MS, methionine synthase; SAHH, S-adenosylhomocysteine hydrolase; MT, methyltransferase.

**Additional file 2**. Table S1. Analytical performance and parameters of the detection of serum methionine metabolites by UHPLC/MS-MS.

## Data Availability

The datasets generated and/or analysed during the current study are not publicly available, since ethics approval and participants’ consent does not allow public sharing of data, but are available from the corresponding author on reasonable request.

## Abbreviations

ALP: Alkaline phosphatase
ALT: Alanine aminotransferase
ANCOVA: Analysis of covariate
ANOVA: Analysis of variance
AST: Aspartate aminotransferase
ATP: Adenosine triphosphate
BMI: Body mass index
CI: Confidence interval
DBP: Diastolic blood pressure
GNHS: Guangzhou Nutrition and Health Study
Hcy: Homocysteine
HDL-C: High-density lipoprotein cholesterol
HOMA-IR: Homoeostasis model assessment of insulin resistance
hsCRP: High-sensitivity C-reactive protein
LDL-C: Low-density lipoprotein cholesterol
LOD: Limit of detection
LOQ: Limit of quantification
MAT: Methionine adenosyltransferase
MET: Metabolic equivalent of task
MS: Methionine synthase
MT: Methyltransferase
NAFLD: Non-alcoholic fatty liver disease
NASH: Non-alcoholic steatohepatitis
OR: Odds ratio
SAH: S-adenosylhomocysteine
SAHH: S-adenosylhomocysteine hydrolase
SAM: S-adenosylmethionine
SBP: Systolic blood pressure
SREBPs: Sterol regulatory element-binding proteins
TC: Total cholesterol
TG: Triglycerides
UA: Uric acid
UHPLC-MS/MS: Ultra-high performance liquid chromatography-tandem mass spectrometry
WHR: Waist-to-hip ratio
