# Supplementary figures and images for: Association of serum methionine metabolites with non-alcoholic fatty liver disease: a cross-sectional study
Source: Nutr Metab (Lond). 2022 Mar 18;19:21. doi: 10.1186/s12986-022-00647-7 (PMC8932073; doi:10.1186/s12986-022-00647-7)

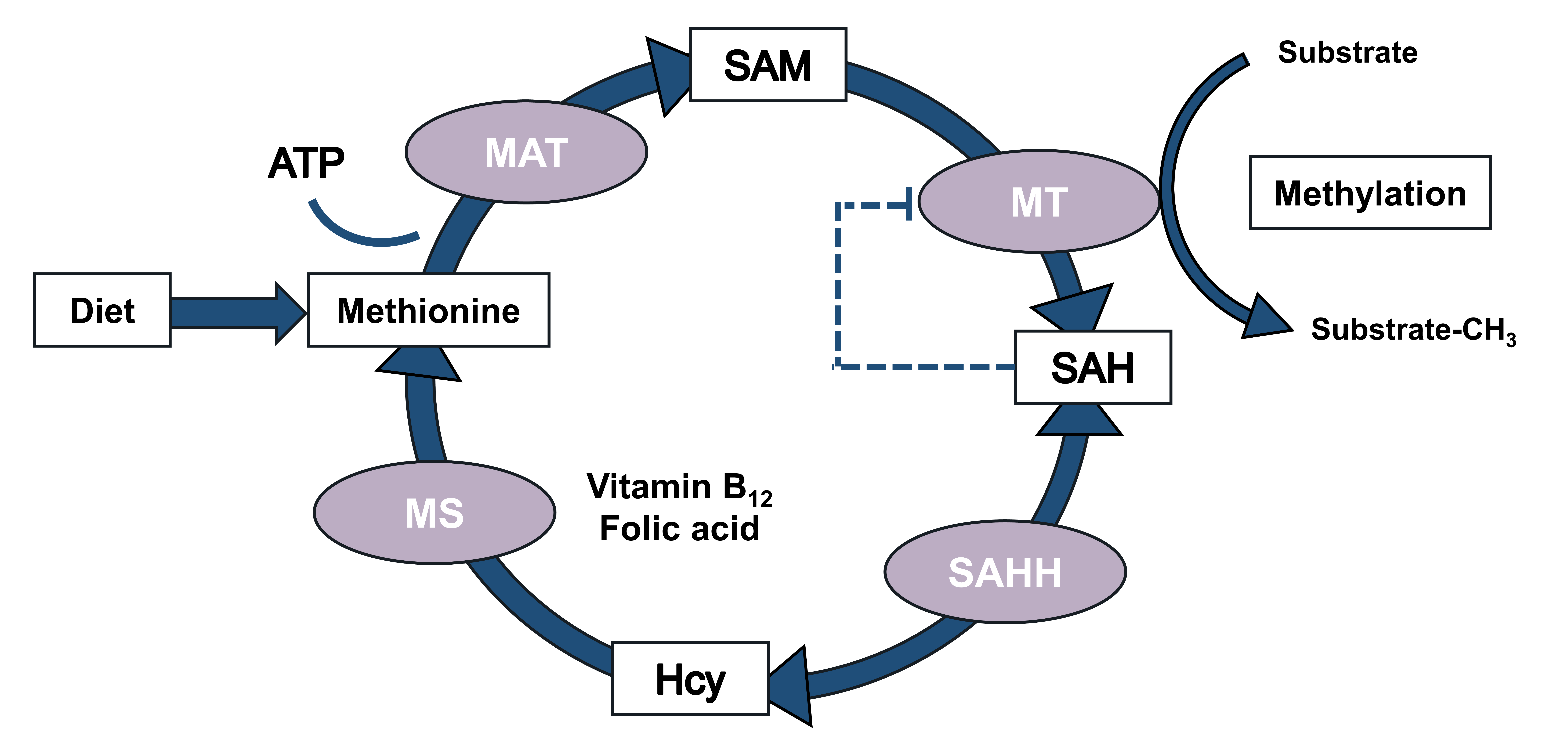

Supplement: Supplementary file 1 — Additional file 1. Figure S1. Methionine metabolism. SAM, S-adenosylmethionine; SAH, S-adenosylhomocysteine; Hcy, homocysteine; ATP, adenosine triphosphate; MAT, methionine adenosyltransferase; MS, methionine synthase; SAHH, S-adenosylhomocysteine hydrolase; MT, methyltransferase. [file 12986_2022_647_MOESM1_ESM.tif]
