# Supplementary material for: Association of serum methionine metabolites with non-alcoholic fatty liver disease: a cross-sectional study
Source: Nutr Metab (Lond). 2022 Mar 18;19:21. doi: 10.1186/s12986-022-00647-7 (PMC8932073; doi:10.1186/s12986-022-00647-7)
Supplement: Supplementary file 2 — Additional file 2. Table S1. Analytical performance and parameters of the detection of serum methionine metabolites by UHPLC/MS-MS. [file 12986_2022_647_MOESM2_ESM.docx]

**Table S1. Analytical performance and parameters of the detection of serum methionine metabolites by UHPLC/MS-MS.**

| **Compound** | **Linear equation** | **R^2^** | **Linear range** | **LOD (nM)** | **LOQ (nM)** | **Recovery (%)** | | | **Intra-day precision (%)** | | | **Inter-day precision (%)** | | |
| --- | --- | --- | --- | --- | --- | --- | --- | --- | --- | --- | --- | --- | --- | --- |
|  |  |  |  |  |  | **Low** | **Medium** | **High** | **Low** | **Medium** | **High** | **Low** | **Medium** | **High** |
| SAM | y=0.6653x+1.6914*10^-4^ | 0.9999 | 2.5–400 nM | 0.1 | 0.05 | 97.9±2.6 | 100.4±2.9 | 99.1±0.8 | 2.9 | 2.3 | 0.5 | 3.6 | 2.0 | 0.4 |
| SAH | y=0.5070x+6.9510*10^-4^ | 0.9999 | 2.5–400 nM | 0.5 | 0.25 | 104.8±2.5 | 103.3±1.2 | 100.5±2.1 | 5.4 | 2.6 | 2.2 | 6.9 | 1.3 | 0.9 |
| Hcy | y=2098.2591x+9.3210*10^-4^ | 0.9974 | 0.0625–10 μM | 14.0 | 7.0 | 103.7±3.4 | 105.6±1.0 | 102.3±0.7 | 5.2 | 2.0 | 2.7 | 1.9 | 4.1 | 0.9 |

LOD, limit of detection; LOQ, limit of quantification.
